# Supplementary material for: M3: Conversational LLMs simplify secure clinical data access, understanding, and analysis
Source: PLOS Digit Health. 2026 Sep 17;5(9):e0001671. doi: 10.1371/journal.pdig.0001671 (PMC13585222; doi:10.1371/journal.pdig.0001671)
Supplement: Supplementary file 1 [file pdig.0001671.s005.pdf]

Dear Dr. Huynh,

We thank the editors and three reviewers for their detailed and constructive feedback. We have revised the manuscript to address all points raised, and our point-by-point responses follow below in the order of the original reviewer comments.

Journal requirements

1. **Financial Disclosure statement.** Will be provided in the submission form with full sentences, grant numbers and organizations, author initials per grant, and a statement on funder role.
2. **Competing Interests.** Added as a dedicated section in the manuscript (see Competing interests section). The authors have no financial relationship with Anthropic or OpenAI and are not employed or compensated by any AI-model provider.
3. **Source file at revision.** Provided as .tex.
4. **Figure files in .tif/.eps.** Will be uploaded separately per PLOS guidelines; figures removed from manuscript file, legends retained.

Reviewer 1

> *"The text has to cite current references."*

Response: We added TrustSQL (Lee et al., 2024; arXiv:2403.15879) to the Benchmarks and evaluation frameworks subsection. This concurrent work formalizes reliability evaluation for text-to-SQL and directly motivates the separate unanswerable-subset evaluation we report.

> *"Include future work in the section on conclusions."*

Response: Conclusion and future work includes an expanded "Roadmap" subsection listing broader dataset coverage, richer MCP tooling, technical enhancements, and ecosystem/community contributions. A new Limitations subsection immediately precedes the Roadmap and frames outstanding gaps as future-work directions.

> *"The manuscript needs to have its typographical errors fixed."*

Response: A typographic and style pass was performed; we corrected a stray character in the Acknowledgments and standardized spelling to US English (e.g., "visualization"). The manuscript was re-read for duplicated words and punctuation consistency.

> *"The current paper has no relation to the prior one."*

Response: We interpret this comment as a request for stronger connection between the manuscript and prior or concurrent literature on reliability-oriented text-to-SQL for clinical data. We addressed this by adding TrustSQL (Lee et al., 2024) to the Benchmarks and evaluation frameworks subsection as closely related concurrent work that formalizes reliability scoring, and by motivating our separate unanswerable-subset evaluation as a direct continuation of that line of research. If the comment referred to a different aspect of the manuscript, we would be glad to address it further once clarified.

> *"Use a succinct and clear message and minimize word repetition."*

Response: The Security and safeguards subsection and the Security, privacy, and accountability subsection were rewritten to tighten claims and remove repeated qualifiers. The Conclusion was likewise trimmed.

> *"Kindly compose your piece as a technical paper, using concise but meaningful sentences. Extraneous components ought to be eliminated."*

Response: We restructured the Methods section into seven labeled subsections (Sampling; Models and deployment; Scoring protocol; Evaluator panel; Statistical analysis; Reproducibility artifacts) to improve scanability and enforce a single concern per paragraph.

> *"Make use of the italicized symbols that are used to separate the texts."*

Response: Code identifiers, table names, column names, and model identifiers are now rendered in \{}texttt{} throughout the manuscript.

> *"The technique section should be shortened for the readers' convenience."*

Response: The Materials and methods section now focuses on design choices rather than narrative exposition; implementation details are referenced to the open-source repository (GitHub) rather than repeated inline.

> *"In the result and discussion section, please emphasize how the acquired findings might be used."*

Response: The Discussion of results subsection and the expanded Limitations subsection together address (a) applicability for exploratory data analysis, (b) the importance of expert oversight for results interpretation, and (c) outstanding gaps that frame where M3 fits into research workflows today.

Reviewer 2

Major concern 1: Evaluation alignment with EHRSQL's reliability framing

> *"The manuscript only evaluates a sample of 100 questions drawn from the answerable subset... The manuscript should either be revised to evaluate the full test set (answerable and unanswerable) using a protocol that is consistent with the intent of the benchmark or be explicitly scoped to answerable-only performance."*

Response: We have taken both steps. The manuscript now:

1. **Scopes the primary quantitative evaluation explicitly to the answerable subset** and identifies it as such in the opening of Evaluation methodology and in the Quantitative performance subsection.
2. **Adds a separate Reliability evaluation on unanswerable questions subsection** (labeled `sec:unanswerable` The gpt-oss-20B condition correctly abstained on 69 of 100 unanswerable questions (69.0%; Wilson 95% CI [59.4%, 77.2%]). The subsection also categorizes the 31 incorrect responses into SQL-backed answers that mapped the question to a mismatched schema element (12 cases), general-knowledge answers produced without abstention (15 cases), and other borderline cases (4). Full conversation transcripts and per-question classifications are included in the M3 GitHub repository alongside the existing answerable transcripts.
3. **Reports the sampling denominators explicitly**: 934 answerable and 233 unanswerable questions in the test set, with random samples of 100 drawn from each (200 evaluated questions in total, Sampling paragraph). We justify n=100 per subset as a trade-off favoring per-question multidisciplinary review over larger unanalyzed samples.

Major concern 2: MCP novelty framing

> *"The manuscript needs to more clearly position the novel aspects, especially those related to MCP."*

Response: We have reframed MCP contribution explicitly. In the Introduction contributions list, contribution (2) now reads: "A concrete, auditable integration of an LLM tool layer with MIMIC-IV using

the Model Context Protocol, providing standardized tool exposure, execution logging, and access-control boundaries. We frame this as an engineering integration of existing technology rather than a novel protocol contribution." We did not conduct a non-MCP-vs-MCP ablation because our contribution is the integration itself rather than a claim about MCP's technical novelty; the reviewer's framing of "auditable integration" is the positioning we adopt.

Major concern 3: Full-scale BigQuery feasibility claims

> *"The 'full-scale BigQuery feasibility' claim should be supported by concrete evidence."*

Response: We have softened this throughout. The contributions list, the Conclusion, and the Data sources and access layer subsection now describe BigQuery support as architectural and identify empirical benchmarking on the full MIMIC-IV v3.1 dataset as future work. No quantitative claims about full-scale performance are made in the revised manuscript.

Major concern 4: Dataset versioning + schema + gpt-oss-20B provenance + contamination

> *"Dataset versioning should be consistent... Statements about the scale of MIMIC-IV should align with PhysioNet's wording, which references certain counts 'as of v3.0.'"*

Response: The Introduction now mirrors PhysioNet's wording exactly: "The current release is MIMIC-IV v3.1; as reported by PhysioNet, as of v3.0 the dataset contains approximately 364,627 unique individuals, 546,028 hospitalizations, and 94,458 ICU stays." The full-dataset citation and demo citation keys were audited: the demo v2.2 reference (Methods) now correctly cites the MIMIC-IV Clinical Database Demo entry on PhysioNet rather than the full-dataset v2.2 entry.

> *"Unless explicitly labeled as benchmark-specific transformations, the SQL examples should reflect standard MIMIC-IV schema conventions (for example, many readers will be surprised by an admissions.age column)."*

Response: Listing 1's caption now explicitly identifies the `admissions.age` column as part of the EHRSQL 2024 benchmark SQLite schema, flagging this as a benchmark-specific transformation.

> *"The description of the open model (gpt-oss-20B) needs stronger provenance reporting and a direct discussion of potential benchmark contamination."*

Response: The Models and deployment paragraph now pins the exact model identifier (`openai/gpt-oss-20b`, released by OpenAI in August 2025 under Apache 2.0 with native MXFP4 quantization; official model card at arXiv:2508.10925) and separately cites the LM Studio community llama.cpp GGUF build that was actually run. Terminology is standardized across the abstract, Methods, cover letter, and Competing Interests: the model is consistently described as "open-weights" (not "open-source"), matching OpenAI's own language. The hosting stack is specified (LM Studio on a MacBook M1 Max with 32 GB RAM), and the open-weights, frozen-parameters nature of the model provides deterministic reproducibility independent of vendor decisions. On contamination, we add a field-level acknowledgement that pretraining exposure to publicly available benchmark artifacts cannot be fully excluded for any modern LLM evaluated against public benchmarks, and pivot to the provision of full conversation transcripts (including tool calls and intermediate reasoning) as our mitigation that enables independent inspection of model behavior on the specific M3 workflow.

Reviewer 3

Major concern 1: Evaluation scope and generalizability of claims

> *"The core empirical evaluation is conducted exclusively on the MIMIC-IV demonstration database (version 2.2, 100-patient subset), not on the full MIMIC-IV dataset. Yet the manuscript makes repeated claims about the system's suitability for 'large-scale research,' 'production-scale deployments,' and 'complex clinical queries.'"*

Response: Agreed. We revised the claims throughout to distinguish between (a) what has been empirically demonstrated (the MIMIC-IV demo via SQLite) and (b) what is architecturally supported (the full MIMIC-IV v3.1 dataset via BigQuery). The phrases "production research," "production-scale deployments," and "highly practical for accelerating research workflows" have been replaced or scoped. The Limitations subsection explicitly names the demo-only empirical scope as the first limitation.

Major concern 2: Statistical analysis

> *"No confidence intervals are reported... The comparison between models is based on a difference of one question... A McNemar test or equivalent paired-sample test is required... No sample size justification is provided."*

Response: All three points have been addressed.

(a) Confidence intervals. The Quantitative performance subsection reports Wilson 95% confidence intervals for both models:

- Claude Sonnet 4 via Claude Desktop: 94/100 = 94.0%, Wilson 95% CI [87.5%, 97.2%]
- gpt-oss-20B: 93/100 = 93.0%, Wilson 95% CI [86.3%, 96.6%]

(b) Paired comparison. We report the full paired 2×2 contingency table (90 both correct; 4 Claude only; 3 gpt-oss-20B only; 3 both wrong) and apply McNemar's test (exact binomial, two-sided) to the 7 discordant pairs. The resulting $p = 1.00$ indicates no statistically detectable difference between models at this sample size. The overlapping confidence intervals are consistent with this conclusion. The Statistical analysis paragraph in Methods states this as the pre-specified analysis.

(c) Sample size rationale. The Sampling paragraph now reports denominators (934 answerable and 233 unanswerable in the EHRSQL 2024 test set) and justifies $n=100$ per subset as a deliberate trade-off favoring depth (per-question multidisciplinary panel review, enabling the per-question error taxonomy) over breadth (narrower confidence intervals on an unanalyzed sample).

Major concern 3: Human evaluation protocol

> *"No details are provided about the evaluation protocol: how many evaluators participated, whether they were blinded to the model identity, what criteria were used to determine semantic equivalence, and how disagreements were resolved."*

Response: Added a dedicated Evaluator panel paragraph in Methods. It specifies:

- Four evaluators drawn from the research group, two with technical expertise (SQL, database schemas, MIMIC-IV structure) and two with clinical domain expertise.
- Each question was reviewed independently by at least two evaluators, including at least one with technical expertise and one with clinical domain expertise.
- Outputs were inspected in their original native formats (Claude Desktop shared conversations for Claude Sonnet 4, locally exported LM Studio JSONs for gpt-oss-20B).

- Disagreements were resolved by discussion among the panel, with the final per-question label reflecting consensus.
- No evaluator has a financial relationship with Anthropic or OpenAI.

Major concern 4: Benchmark sampling strategy

> *"The authors should report the total size of the answerable test subset, justify the 100-question sample, and discuss how the exclusion of unanswerable questions may affect the generalizability of the reported accuracy figures."*

Response: All three items are addressed. The Sampling paragraph reports 934 answerable and 233 unanswerable questions in the test set. The n=100 per subset justification is given as above (depth over breadth). The potential generalizability impact is addressed in two places: (a) the new Reliability evaluation on unanswerable questions subsection directly measures unanswerable behavior rather than extrapolating, and (b) the Limitations subsection notes the demo-scope of the evaluation.

Major concern 5: Undeclared potential conflict of interest

> *"The manuscript does not address this relationship in the competing interests section."*

Response: A new Competing interests section has been added immediately before the Acknowledgments. It discloses that MCP is developed by Anthropic, that Claude Sonnet 4 is also developed by Anthropic, and that gpt-oss-20B is an open-weights model released by OpenAI. The authors have no financial relationship with either Anthropic or OpenAI and are not employed or compensated by any AI-model provider.

Minor concern 6: Temporal simulation methodology

Response: The Models and deployment paragraph now includes a one-sentence acknowledgement that some temporal-reasoning failures reported in the error analysis may reflect prompt sensitivity to the unusual timestamp rather than fundamental temporal-reasoning limitations.

Minor concern 7: Systematic error taxonomy

Response: Added Table (Error taxonomy) summarizing the 10 error-producing questions across four categories (Question ambiguity/underspecification; Schema exploration gap; Temporal logic; Frequency tie-breaking), broken down by Shared/Claude-only/gpt-oss-20B-only/Total. The pattern matches the narrative: ambiguity dominates the distribution (5/10) and accounts for all three shared failures.

Minor concern 8: gpt-oss-20B model identifier

Response: The Models and deployment paragraph now pins the full identifier (`openai/gpt-oss-20b`), hosting stack (LM Studio), release provenance (OpenAI, August 2025, arXiv:2508.10925), and native MXFP4 quantization, and separately cites the specific LM Studio community GGUF build that was run. Because the model has open, frozen weights, re-runs are exactly reproducible given identical prompts.

Minor concern 9: Security claims and empirical validation

> *"Phrases such as 'robust protection,' 'sophisticated syntactic analysis,' and 'comprehensive security measures' present as stronger assurances than the evidence supports."*

Response: All instances of these qualifiers have been removed or replaced with concrete descriptions (sqlparse-based validator; per-user request-count rate limiter; OAuth2/JWT tokens). A single sentence at the opening of Security and safeguards identifies formal security evaluation (adversarial SQL-injection testing, penetration testing, authentication audits) as future work. We did not conduct penetration testing for this submission.

Minor concern 10: Related work coverage

> *"Recent LLM-based text-to-SQL systems that have been applied to MIMIC or similar clinical datasets... RAG-based approaches to clinical QA... existing MCP-based tools in the biomedical domain beyond the two non-peer-reviewed blog posts currently cited."*

Response: We added TrustSQL (Lee et al., 2024) to the Benchmarks and evaluation frameworks subsection as the closest concurrent work on text-to-SQL reliability evaluation, and MedRAG (Xiong et al., 2024) to the Natural language interfaces for medical data subsection as a complementary RAG-over-biomedical-corpora benchmark that grounds answers in retrieved documents rather than structured databases. We retain the existing MCP adoption references to illustrate that MCP is being applied beyond software development, and note that peer-reviewed MCP-in-biomedicine literature is nascent given MCP's November 2024 release.

Minor concern 11: Limitations section

Response: Added a dedicated Limitations subsection in Conclusion and future work. It covers: (a) empirical evaluation restricted to the MIMIC-IV demo subset; (b) risk of SQL queries that execute successfully while reflecting an incorrect interpretation of the question, and the role of exposed SQL for expert review; (c) absence of end-user studies with practicing clinical researchers (planned future work); (d) institutional-deployment considerations for authentication and governance.

Minor concern 12: Listing 1 translation artifact

> *"The SQL query in Listing 1 appears to have been machine-translated into Spanish."*

Response: The translation artifact was introduced by the reviewer's PDF rendering tool and was not present in our submission. The Listing 1 SQL in our manuscript source uses standard (English) SQL keywords. We confirm this for the revision submission.

We thank the reviewers again for their thorough and constructive feedback. The paired statistical analysis (McNemar's test yielding $p = 1.00$) and the paired 2×2 contingency table (90 both correct, 4 Claude-only, 3 gpt-oss-only, 3 both wrong) were directly motivated by Reviewer 3's statistical critique and, in our view, strengthen the manuscript considerably by replacing a qualitative "comparable" judgment with a quantitative one.

Sincerely,

Rafi Al Attrach, Pedro Moreira, Rajna Fani, Renato Umeton, Amelia Fiske, and Leo Anthony Celi

Corresponding author: Leo Anthony Celi – lceli@mit.edu
