# Supplementary material for: M3: Conversational LLMs simplify secure clinical data access, understanding, and analysis
Source: PLOS Digit Health. 2026 Sep 17;5(9):e0001671. doi: 10.1371/journal.pdig.0001671 (PMC13585222; doi:10.1371/journal.pdig.0001671)
Supplement: Supplementary file 2 [file pdig.0001671.s006.pdf]

Response to Reviewers

Manuscript PDIG-D-25-00875R1
M3: Conversational LLMs simplify secure clinical data access, understanding, and analysis
PLOS Digital Health

Dear Dr. Huynh,

We thank you and the three reviewers for the second review. Reviewer 1 indicated that all earlier comments were addressed, and Reviewers 2 and 3 raised a small set of clarifications on scope, reproducibility, BigQuery, security wording, and the unanswerable evaluation. We have made the corresponding edits. None of them changes the core findings: 94 and 93 percent accuracy on the sampled answerable questions, McNemar exact test $p = 1.00$, and 69 percent correct abstention for gpt-oss-20B on the sampled unanswerable questions. The changes sharpen what is claimed and make the open-weights run easier to reproduce. Our point-by-point replies follow, in the order of the reviewer comments. Section names refer to the revised manuscript.

Reviewer 1

Reviewer 1 marked all comments as addressed in the review form. We also went through the repeated free-text items and made a final technical writing pass.

Cite current references.

Response. We reviewed the reference set. It already draws on recent work in clinical text-to-SQL and clinical question answering: the EHRSQL 2024 shared task, TrustSQL (2024), BiomedSQL (2025), MedRAG (2024), the MIMIC-IV v3.1 release, and the gpt-oss-20B model card (2025). We added one further current reference, a 2025 deployment-centric analysis of gpt-oss-20B (Kumar et al.), in *Models and deployment*, where it supports the local-deployment results.

Include future work in the conclusion.

Response. The Conclusion already contained a Limitations subsection and a Roadmap. We tightened it so that the empirical scope (the MIMIC-IV demonstration database in the EHRSQL 2024 setting) leads, and the future-work items are explicit: full MIMIC-IV evaluation through the BigQuery backend, formal security testing, end-user studies, richer identity-provider integration, and ambiguity handling before SQL generation.

Fix typographical errors and keep a concise technical style.

Response. We made another copyedit pass across the abstract, author summary, related work, methods, discussion, ethics, and conclusion. We removed repeated qualifiers, shortened broad phrasing, and replaced the few promotional adjectives with concrete implementation details. We also standardized punctuation and removed the dashes that remained in the previous version.

Relation to prior work.

Response. The manuscript positions M3 against prior and concurrent clinical text-to-SQL work (EHRSQL, TrustSQL, BiomedSQL, MedRAG), MIMIC-IV access tooling, and MCP-based integration, and states that its contribution is an auditable engineering integration rather than a new protocol.

Use italicized or set-apart symbols for identifiers.

Response. Table names, column names, model identifiers, and SQL artifacts are set in monospace where they denote code or schema elements, which distinguishes them from running text. We extended this formatting to the identifiers that remained in plain text in the error analysis.

Shorten the technique section and show how the findings are used.

Response. Methods keeps the labeled-subsection structure introduced in the previous revision. We added a concrete statement in *Discussion of results* of how the current findings are used today: exploratory data analysis, inspecting and teaching the SQL behind a clinical question, and drafting queries that a domain expert reviews before use.

Reviewer 2

Abstention was evaluated only for gpt-oss-20B.

> *Statements such as "both models answered 93 to 94% of clinical questions correctly" should be narrowed to "sampled answerable benchmark questions."*

Response. Agreed. The abstract and author summary now tie the 93 to 94 percent figure to a sample of answerable benchmark questions, and the abstract states that abstention was measured only for gpt-oss-20B. The author summary and the *Reliability evaluation on unanswerable questions* section state explicitly that we did not run the unanswerable evaluation on Claude Sonnet 4 and make no claim about its abstention behavior.

BigQuery support should be described as architectural, not demonstrated.

Response. We rewrote the BigQuery paragraph in *Data sources and access layer*. It now states that the backend is provided as architectural support, and that BigQuery-specific SQL generation, SQLite-to-BigQuery dialect differences, cost-control behavior, IAM enforcement, query latency, and runtime performance at full-dataset scale were not part of the empirical evaluation, which used the local SQLite demonstration setting. The abstract and Conclusion state that full-dataset BigQuery evaluation is future work.

The reproducibility claim is too strong.

Response. We removed the phrase "deterministic reproducibility" wherever it appeared. The manuscript now states that the open, frozen weights of gpt-oss-20B make the condition repeatable, while exact replication also depends on matching the prompts, tool definitions, decoding settings, software and quantization build, and runtime configuration. We added the concrete settings in *Models and deployment*: we did not tune decoding and used the values LM Studio applied for the model build (temperature 0.8, top-p 0.8, top-k 40, minimum-p 0.05, repeat penalty 1.1, 32,000-token context) with a fixed random seed of 3.

Reviewer 3

Security wording.

Please keep security claims strictly implementation-descriptive (OAuth2/JWT, read-only validation via sqlparse, rate limiting, output limits, audit logging) and avoid guarantee-style adjectives unless you provide testing evidence.

Response. We removed the guarantee-style adjectives (robust, comprehensive, sophisticated, and similar) where they described M3's own capabilities, in the abstract, author summary, related work, methods, ethics, and conclusion. The security text is now implementation-descriptive: OAuth2 and JWT based access control, read-only validation through sqlparse, per-user rate limiting, output-size limits, and audit logging. *Security and safeguards* and the Conclusion continue to state that formal security testing (adversarial SQL-injection testing, penetration testing, and authentication audits) is future work.

Scope in abstract and conclusion.

Response. Both sections now state the empirical scope. The abstract reports that performance is characterized on the MIMIC-IV demonstration database in the EHRSQL 2024 setting and that full-dataset BigQuery evaluation remains future work. The Conclusion opens by scoping the result to the demonstration setting and lists full MIMIC-IV evaluation through BigQuery as future work, so readers cannot read the figures as large-scale validated performance.

Unanswerable subset evaluated only with gpt-oss-20B; clarify abstention.

Response. The *Reliability evaluation on unanswerable questions* section now states that Claude Sonnet 4 was not evaluated on the unanswerable subset and that we make no claim about its abstention behavior. It also clarifies that abstention is the model's own behavior under the M3 prompt and tool setup: M3 does not enforce a separate rule that decides when to decline, so the outcome reflects how the model responds rather than a system-level policy.

Reproducibility details.

Response. The decoding settings are now reported in *Models and deployment*, as described under Reviewer 2 above: the untuned LM Studio values for the model build (temperature 0.8, top-p 0.8, top-k 40, minimum-p 0.05, repeat penalty 1.1, 32,000-token context) with a fixed random seed of 3. Full conversation transcripts for both subsets, including tool calls and intermediate reasoning, are in the M3 GitHub repository.

We thank the reviewers again for a careful read. We believe the revised manuscript states its scope precisely and reports the open-weights evaluation in enough detail for others to reproduce it.

Sincerely,

Rafi Al Attrach, Pedro Moreira, Rajna Fani, Renato Umeton, Amelia Fiske, and Leo Anthony Celi
Corresponding author: Leo Anthony Celi (lceli@mit.edu)
