# Supplementary material for: M3: Conversational LLMs simplify secure clinical data access, understanding, and analysis
Source: PLOS Digit Health. 2026 Sep 17;5(9):e0001671. doi: 10.1371/journal.pdig.0001671 (PMC13585222; doi:10.1371/journal.pdig.0001671)
Supplement: Peer review history

# Peer review: Decision Letter 0

**DOI:** 10.1371/journal.pdig.0001671.r001

**Authors:** Phat Huynh, Zhenwei Shi

23 Feb 2026

Response to Reviewers Revised Manuscript with Track Changes Manuscript **Journal Requirements:**

1. Please amend your detailed Financial Disclosure statement. This is published with the article. It must therefore be completed in full sentences and contain the exact wording you wish to be published.

i. Please clarify all sources of financial support for your study. List the grants, grant numbers, and organizations that funded your study, including funding received from your institution. Please note that suppliers of material support, including research materials, should be recognized in the Acknowledgements section rather than in the Financial Disclosure.

ii. State the initials, alongside each funding source, of each author to receive each grant. For example: "This work was supported by the National Institutes of Health (####### to AM; ###### to CJ) and the National Science Foundation (###### to AM)."

iii. State what role the funders took in the study. If the funders had no role in your study, please state: “The funders had no role in study design, data collection and analysis, decision to publish, or preparation of the manuscript.”

iv. If any authors received a salary from any of your funders, please state which authors and which funders.

2. Please send a completed 'Competing Interests' statement, including any COIs declared by your co-authors. If you have no competing interests to declare, please state "The authors have declared that no competing interests exist". Otherwise please declare all competing interests beginning with the statement "I have read the journal's policy and the authors of this manuscript have the following competing interests:"

3. We ask that a manuscript source file is provided at Revision. Please upload your manuscript file as a .doc, .docx, .rtf or .tex.

4. Please upload separate figure files in .tif or .eps format. Also, remove the figures from your manuscript file but keep the legends.

For more information about figure files please see our guidelines:

https://journals.plos.org/globalpublichealth/s/figures

https://journals.plos.org/globalpublichealth/s/figures#loc-file-requirements

**Additional Editor Comments (if provided):** **Reviewers' Comments:**

**Comments to the Author**

1. Does this manuscript meet PLOS Digital Health’s publication criteria?

Reviewer #1: Yes

Reviewer #2: Partly

Reviewer #3: Partly

**********

2. Has the statistical analysis been performed appropriately and rigorously?

Reviewer #1: Yes

Reviewer #2: No

Reviewer #3: No

**********

3. Have the authors made all data underlying the findings in their manuscript fully available (please refer to the Data Availability Statement at the start of the manuscript PDF file)?

The PLOS Data policy

Reviewer #1: Yes

Reviewer #2: Yes

Reviewer #3: Yes

**********

4. Is the manuscript presented in an intelligible fashion and written in standard English?

Reviewer #1: Yes

Reviewer #2: Yes

Reviewer #3: Yes

**********

Reviewer #1: The text has to cite current references.

• Include future work in the section on conclusions.

• The manuscript needs to have its typographical errors fixed.

• The current paper has no relation to the prior one.

• Use a succinct and clear message and minimize word repetition.

• Kindly compose your piece as a technical paper, using concise but meaningful sentences. Extraneous components ought to be eliminated.

Kindly write it like a report for an article.

• Make use of the italicized symbols that are used to separate the texts.

• The technique section should be shortened for the readers' convenience because it includes too little information about the equipment's use.

• In the result and discussion section, please emphasize how the acquired findings might be used. More information is needed.

Reviewer #2: The manuscript presents M3, a conversational interface that translates natural-language questions into SQL for MIMIC-IV and returns results with the generated query, supporting both a local SQLite demo workflow and a BigQuery workflow. The topic is important and the system appears practically motivated, but the current version does not yet provide sufficiently comparable, reproducible, and verifiable evidence for its key claims (benchmark performance, secure access, and full-scale feasibility).

Major concerns

・ The main issues are evaluation alignment and interpretability. Although EHRSQL 2024 is presented as a "reliable" text-to-SQL task, in which systems should respond with confidence and refrain from answering when uncertain, including on unanswerable questions, the manuscript only evaluates a sample of 100 questions drawn from the answerable subset. It then reports a single "accuracy" number based on manual comparison. This makes the reported performance difficult to compare to shared-task results and risks overstating reliability in settings where recognizing unanswerability is important. The manuscript should either be revised to evaluate the full test set (answerable and unanswerable) using a protocol that is consistent with the intent of the benchmark or be explicitly scoped to answerable-only performance. In the latter case, the paper should characterize unanswerable behavior as a limitation and report how the system behaves in those cases.

・ The manuscript needs to more clearly position the novel aspects, especially those related to MCP. If MCP is an implementation choice, the paper should explicitly frame its contribution as the concrete and auditable integration of an LLM tool layer with MIMIC-IV. It should emphasize the operational benefits, such as standardized tool exposure, logging, and boundary setting. If the authors claim that MCP is technically essential, they should provide a comparison or ablation study against a non-MCP tool-calling baseline to demonstrate the measurable changes that MCP brings to reliability, auditability, security controls, developer effort, and portability. As it stands, readers may view MCP as interchangeable infrastructure rather than a core contribution, unless its incremental value is stated and evidenced more directly.

・ The "full-scale BigQuery feasibility" claim should be supported by concrete evidence. It should also explain how the system handles BigQuery-specific SQL differences and operational constraints. Without these measurements and controls, the BigQuery discussion reads more as intended design than demonstrated capability, especially since the quantitative benchmark results appear tied to the demo SQLite setting.

・ Dataset versioning should be consistent across the system description and the data availability statement. Statements about the scale of MIMIC-IV should align with PhysioNet’s wording, which references certain counts "as of v3.0." Unless explicitly labeled as benchmark-specific transformations, the SQL examples should reflect standard MIMIC-IV schema conventions (for example, many readers will be surprised by an admissions.age column). Given that benchmark artifacts and the MIMIC demo subset are broadly accessible, the description of the open model (gpt-oss-20B) needs stronger provenance reporting and a direct discussion of potential benchmark contamination.

Reviewer #3: General Assessment

This manuscript presents M3, a natural language interface for querying the MIMIC-IV clinical database via the Model Context Protocol (MCP). The system addresses a genuine and relevant problem in clinical informatics: reducing the technical barriers that prevent clinical researchers without SQL expertise from leveraging large-scale EHR datasets. The paper is clearly written, the system architecture is reasonably described, and the open-source availability of both the code and benchmark results is commendable. However, several methodological limitations must be addressed before the manuscript can be considered for publication.

Major Concerns

1. Evaluation scope and generalizability of claims

The core empirical evaluation is conducted exclusively on the MIMIC-IV demonstration database (version 2.2, 100-patient subset), not on the full MIMIC-IV dataset. Yet the manuscript makes repeated claims about the system's suitability for "large-scale research," "production-scale deployments," and "complex clinical queries." These claims are not supported by the evidence presented.

The authors describe BigQuery integration with the full MIMIC-IV v3.1 dataset as a key feature of M3, but no empirical evaluation is provided for this backend. Performance on a 100-patient SQLite demo may differ substantially from the full dataset in terms of query complexity, schema coverage, and response correctness. The authors must either (a) provide empirical evaluation on the full MIMIC-IV dataset via BigQuery, or (b) substantially revise all claims about large-scale suitability to accurately reflect that the evaluation is limited to the demo subset.

2. Statistical analysis is absent

The results section reports accuracy as simple percentages (94% for Claude Sonnet 4, 93% for gpt-oss-20B) without any inferential statistics. This is insufficient for a research article making quantitative performance claims. Specifically:

No confidence intervals are reported for the accuracy estimates. With n=100, the 95% CI for a proportion of 0.94 is approximately [0.87–0.98], a range wide enough to substantially qualify the precision of the reported results.

The comparison between models is based on a difference of one question (94 vs. 93 correct answers) with no statistical test applied. The authors conclude that gpt-oss-20B performance is "comparable" to Claude Sonnet 4, but this conclusion is statistically unsupported. A McNemar test or equivalent paired-sample test is required for this comparison.

No sample size justification is provided. The selection of 100 questions from a larger benchmark test set is not accompanied by a power calculation or a rationale demonstrating that this sample is sufficient to estimate performance with adequate precision or to detect meaningful differences between models.

The authors must provide: (a) confidence intervals for all reported accuracy metrics, (b) an appropriate statistical test for the between-model comparison, and (c) a justification for the chosen sample size.

3. Human evaluation protocol lacks rigor and reproducibility

The manuscript states that answer correctness was determined by human judgment when automated evaluation was insufficient. However, no details are provided about the evaluation protocol: how many evaluators participated, whether they were blinded to the model identity, what criteria were used to determine semantic equivalence, and how disagreements were resolved. Without this information, the evaluation cannot be considered reproducible or free from assessor bias.

This is particularly important given that the system under evaluation is built on Anthropic's MCP and that one of the two models evaluated — the top performer — is Claude Sonnet 4, also from Anthropic. While the authors' institutional affiliations do not necessarily constitute a conflict of interest, the evaluation design must be sufficiently rigorous to rule out unintentional bias. The authors should: (a) fully describe the human evaluation protocol, (b) report inter-rater agreement metrics (e.g., Cohen's kappa) if more than one evaluator was involved, and (c) clarify the affiliations and potential interests of those who performed the evaluation.

4. Benchmark sampling strategy introduces potential bias

The authors selected 100 questions from the "answerable" subset of the EHRSQL 2024 test set, explicitly excluding unanswerable questions. This filtering may inflate the reported accuracy relative to real-world usage conditions, where researchers will inevitably pose questions that are ambiguous, malformed, or unanswerable given the available data. The manuscript does not report the total size of the answerable subset from which the 100 questions were drawn, nor does it justify why random sampling from this subset — rather than using the full answerable set — was chosen.

The authors should report the total size of the answerable test subset, justify the 100-question sample, and discuss how the exclusion of unanswerable questions may affect the generalizability of the reported accuracy figures.

5. Undeclared potential conflict of interest

M3 is built upon Anthropic's Model Context Protocol, and the best-performing model in the evaluation is Claude Sonnet 4, a proprietary model developed by Anthropic. The manuscript does not address this relationship in the competing interests section. Even if no financial relationship exists between the authors and Anthropic, readers and editors are entitled to a transparent statement acknowledging that the evaluation involves a model from the same company whose protocol underpins the entire system. The authors should add an explicit statement to the competing interests section addressing this point.

Minor Concerns

6. Temporal simulation methodology requires clarification

The authors simulate a fixed current timestamp ("2100-12-31 23:59:00") by prepending a contextual instruction to each prompt, in order to align with the EHRSQL 2024 benchmark requirements. While this is described, it is not sufficiently discussed as a potential source of evaluation artefact. It is unclear whether this approach reliably conditions model behavior across all temporal query types, or whether some query failures attributed to "complex temporal reasoning" may in fact reflect prompt sensitivity to this unusual timestamp. A brief analysis or at least an explicit acknowledgement of this limitation would strengthen the methods section.

7. Error analysis lacks systematic categorization

The qualitative error analysis identifies some interesting failure patterns (temporal ambiguity, implicit clinical assumptions, schema-specific knowledge gaps), but does not provide a systematic taxonomy of error types with frequency counts. The manuscript would benefit from a structured error categorization table showing the distribution of failure modes across both models, which would make the analysis more informative and reproducible.

8. The "gpt-oss-20B" model identifier is non-standard

The manuscript refers to the open-source model as "gpt-oss-20B" without providing sufficient identifying information. The reference (citation 29) points to a HuggingFace repository hosted by lmstudio-community, but does not clarify the original model family, training data, or provenance. Given that reproducibility is a stated goal of the system, the authors should provide the full model identifier, version, quantization level (GGUF format is mentioned), and any relevant model card information. If this model is a derivative or fine-tuned version of an existing model, that relationship should be made explicit.

9. Security claims require empirical validation

The manuscript makes substantive claims about M3's security architecture, including OAuth2 authentication, SQL validation, rate limiting, and audit logging. These claims are described architecturally but are not supported by any security testing or formal evaluation. Phrases such as "robust protection," "sophisticated syntactic analysis," and "comprehensive security measures" present as stronger assurances than the evidence supports. The authors should either (a) provide results from security testing (e.g., SQL injection attempts, access control validation), or (b) qualify these claims as design intentions pending formal security evaluation.

10. Related work coverage has notable gaps

The related work section covers the evolution of NLIDB systems and clinical benchmarks reasonably well, but does not engage with several relevant recent developments. Specifically, the authors should discuss: (a) recent LLM-based text-to-SQL systems that have been applied to MIMIC or similar clinical datasets beyond those already cited, (b) RAG-based approaches to clinical QA that partially overlap with M3's intended use case, and (c) existing MCP-based tools in the biomedical domain beyond the two non-peer-reviewed blog posts currently cited (references 26 and 27). Citations 26 and 27 are industry blog posts rather than peer-reviewed sources; these should be replaced or supplemented with more rigorous references.

11. Limitations section is underdeveloped

The limitations discussed in the manuscript are brief and concentrated in the conclusion. The authors acknowledge the focus on MIMIC-IV and the dependence on LLM quality, but do not discuss: (a) the risk of hallucinated SQL queries that appear syntactically valid but are semantically incorrect in ways that non-expert users may not detect, (b) the implications of using a 100-patient demo dataset for evaluation, (c) the lack of evaluation on actual clinical researchers as end users (usability evaluation is entirely absent), and (d) potential failure modes in institutional deployment contexts where authentication and data governance requirements may differ from those assumed in the current design. A more comprehensive limitations section is necessary.

12. Figure and listing presentation

The SQL query in Listing 1 appears to have been machine-translated into Spanish (the document reviewed was a translated version), which introduces errors in SQL syntax — for instance, SQL keywords such as SELECT, FROM, JOIN, WHERE, and GROUP BY appear translated as SELECCIONAR, DESDE, UNIÓN, DONDE, and GRUPO POR. The authors should ensure that all code listings in the final submission appear in their original, untranslated form, as SQL is a language-independent standard and translation of keywords renders the code non-functional and misleading.

Summary of Recommended Actions

The manuscript describes a promising and practically motivated system, but requires substantial revision before it can meet PLOS Digital Health's criteria for methodological rigor. The following actions are necessary:

The authors must provide inferential statistics (confidence intervals and between-model comparison tests) for all quantitative results. The human evaluation protocol must be described in full, including evaluator details and inter-rater agreement. Claims about large-scale and production-ready performance must be revised to accurately reflect the demo-scale evaluation, or supplemented with full-scale empirical results. The benchmark sampling strategy must be justified and its implications for generalizability discussed. The competing interests statement must explicitly address the relationship between M3, MCP, and Claude Sonnet 4. Security claims must be qualified as design features rather than validated properties. The limitations section must be substantially expanded.

**********

what does this mean?). If published, this will include your full peer review and any attached files.

**Do you want your identity to be public for this peer review?**  If you choose “no”, your identity will remain anonymous but your review may still be made public.

For information about this choice, including consent withdrawal, please see our Privacy Policy

Reviewer #1: No

Reviewer #2: No

Reviewer #3: **Yes:** Carlos Luis Parra-Calderón

**********

**Figure resubmission:**

**Reproducibility:**

To enhance the reproducibility of your results, we recommend that authors of applicable studies deposit laboratory protocols in protocols.io, where a protocol can be assigned its own identifier (DOI) such that it can be cited independently in the future. Additionally, PLOS ONE offers an option to publish peer-reviewed clinical study protocols. Read more information on sharing protocols at https://plos.org/protocols?utm_medium=editorial-email&utm_source=authorletters&utm_campaign=protocols

# Author comment: Author response to Decision Letter 1

**DOI:** 10.1371/journal.pdig.0001671.r002

22 Apr 2026

Attachment Submitted filename: Response_to_Reviewers.pdf

# Peer review: Decision Letter 1

**DOI:** 10.1371/journal.pdig.0001671.r003

**Authors:** Phat Huynh, Zhenwei Shi

22 May 2026

Response to ReviewersRevised Manuscript with Track ChangesManuscript**Journal Requirements:** **Additional Editor Comments (if provided):** **Reviewers' Comments:**

**Comments to the Author**

Reviewer #1: All comments have been addressed

Reviewer #2: (No Response)

Reviewer #3: (No Response)

**********

publication criteria?

Reviewer #1: Yes

Reviewer #2: Yes

Reviewer #3: Partly

**********

3. Has the statistical analysis been performed appropriately and rigorously?

Reviewer #1: Yes

Reviewer #2: Yes

Reviewer #3: Yes

**********

4. Have the authors made all data underlying the findings in their manuscript fully available (please refer to the Data Availability Statement at the start of the manuscript PDF file)?

The PLOS Data policy

Reviewer #1: Yes

Reviewer #2: Yes

Reviewer #3: Yes

**********

5. Is the manuscript presented in an intelligible fashion and written in standard English?

Reviewer #1: Yes

Reviewer #2: Yes

Reviewer #3: Yes

**********

Reviewer #1: The text has to cite current references.

• Include future work in the section on conclusions.

• The manuscript needs to have its typographical errors fixed.

• The current paper has no relation to the prior one.

• Use a succinct and clear message and minimize word repetition.

• Kindly compose your piece as a technical paper, using concise but meaningful sentences. Extraneous components ought to be eliminated.

Kindly write it like a report for an article.

• Make use of the italicized symbols that are used to separate the texts.

• The technique section should be shortened for the readers' convenience because it includes too little information about the equipment's use.

• In the result and discussion section, please emphasize how the acquired findings might be used. More information is needed.

Reviewer #2: The revised manuscript is substantially improved and addresses most of my previous concerns. However, several clarifications are still needed before acceptance.

First, the EHRSQL reliability issue is mostly addressed, but the unanswerable-question evaluation is reported only for gpt-oss-20B. The manuscript should therefore avoid implying that abstention behavior has been evaluated for both models. Statements such as “both models answered 93–94% of clinical questions correctly” should be narrowed to “sampled answerable benchmark questions.”

Second, the BigQuery claims have been softened, but the manuscript should more explicitly state that BigQuery-specific SQL generation, dialect differences, cost controls, IAM behavior, latency, and runtime performance were not empirically evaluated. BigQuery support should be described as architectural support rather than demonstrated full-scale feasibility.

Third, the dataset and model provenance issues are improved. The manuscript now distinguishes the MIMIC-IV demo v2.2 benchmark setting from the full MIMIC-IV v3.1 dataset, and it identifies admissions.age as a benchmark-specific schema transformation. The gpt-oss-20B description is also clearer. However, the reproducibility claim remains too strong: open weights and frozen parameters improve reproducibility, but they do not guarantee exact replication without identical prompts, decoding settings, software versions, quantization build, and runtime configuration.

Reviewer #3: Thank you for the revision. Many of the key concerns from the previous round are now adequately addressed. In particular, you added appropriate statistical reporting (Wilson 95% CIs) and a paired comparison using McNemar on the matched 100 answerable questions, which fixes the earlier “93 vs 94” interpretation problem. You also clarified the human evaluation protocol (multidisciplinary panel, independent review by at least two evaluators per item, consensus resolution), which significantly improves reproducibility and trust in the labels. Finally, the scope is better aligned: you now distinguish what is empirically evaluated (demo SQLite / EHRSQL setup) from what is only architecturally supported (BigQuery/full dataset), and you state this as a limitation.

What still needs to be fixed (minor revision items):

Security wording: even though you note formal security testing is future work, some language still reads like assurance (“robust/comprehensive/sophisticated”). Please keep security claims strictly implementation-descriptive (OAuth2/JWT, read-only validation via sqlparse, rate limiting, output limits, audit logging) and avoid guarantee-style adjectives unless you provide testing evidence.

Scope in abstract/conclusion: please ensure the abstract and conclusion consistently state that empirical evaluation is on the MIMIC-IV demo / EHRSQL setting, while full-scale BigQuery evaluation remains future work, so readers cannot over-infer “large-scale validated performance.”

Unanswerable subset evaluated only with gpt-oss-20B: add one explicit sentence that you do not claim the same abstention profile would hold for Claude without direct testing, and clarify whether abstention is a model behaviour or a system policy.

Reproducibility details: you mention “deterministic reproducibility” for gpt-oss-20B; please report decoding settings (temperature/top_p/max tokens) and any determinism-related configuration so others can replicate precisely.

**********

what does this mean?). If published, this will include your full peer review and any attached files.

**Do you want your identity to be public for this peer review?**  If you choose “no”, your identity will remain anonymous but your review may still be made public.

For information about this choice, including consent withdrawal, please see our Privacy Policy

Reviewer #1: No

Reviewer #2: No

Reviewer #3: **Yes:** Carlos Luis Parra-Calderón

**********

**Figure resubmission:**

**Reproducibility:**

To enhance the reproducibility of your results, we recommend that authors of applicable studies deposit laboratory protocols in protocols.io, where a protocol can be assigned its own identifier (DOI) such that it can be cited independently in the future. Additionally, PLOS ONE offers an option to publish peer-reviewed clinical study protocols. Read more information on sharing protocols at https://plos.org/protocols?utm_medium=editorial-email&utm_source=authorletters&utm_campaign=protocols

# Author comment: Author response to Decision Letter 2

**DOI:** 10.1371/journal.pdig.0001671.r004

28 Jun 2026

Attachment Submitted filename: Response_to_Reviewers_R2.pdf

# Peer review: Decision Letter 2

**DOI:** 10.1371/journal.pdig.0001671.r005

**Authors:** Phat Huynh, Zhenwei Shi

3 Aug 2026

M3: Conversational LLMs simplify secure clinical data access, understanding, and analysis

PDIG-D-25-00875R2

Dear Dr. Celi,

We are pleased to inform you that your manuscript 'M3: Conversational LLMs simplify secure clinical data access, understanding, and analysis' has been provisionally accepted for publication in PLOS Digital Health.

Before your manuscript can be formally accepted you will need to complete some formatting changes, which you will receive in a follow-up email from a member of our team.

Please note that your manuscript will not be scheduled for publication until you have made the required changes, so a swift response is appreciated.

IMPORTANT: The editorial review process is now complete. PLOS will only permit corrections to spelling, formatting or significant scientific errors from this point onwards. Requests for major changes, or any which affect the scientific understanding of your work, will cause delays to the publication date of your manuscript.

If your institution or institutions have a press office, please notify them about your upcoming paper to help maximize its impact. If they'll be preparing press materials, please inform our press team as soon as possible -- no later than 48 hours after receiving the formal acceptance. Your manuscript will remain under strict press embargo until 2 pm Eastern Time on the date of publication. For more information, please contact digitalhealth@plos.org.

Thank you again for supporting Open Access publishing; we are looking forward to publishing your work in PLOS Digital Health.

Best regards,

Phat Kim Huynh, Ph.D.

Guest Editor

PLOS Digital Health

***********************************************************

**Additional Editor Comments (if provided):**

**Reviewer Comments (if any, and for reference):**

Reviewer's Responses to Questions

**Comments to the Author**

Reviewer #1: All comments have been addressed

Reviewer #2: All comments have been addressed

Reviewer #3: All comments have been addressed

**********

publication criteria?

Reviewer #1: Yes

Reviewer #2: Yes

Reviewer #3: Yes

**********

3. Has the statistical analysis been performed appropriately and rigorously?

Reviewer #1: Yes

Reviewer #2: N/A

Reviewer #3: Yes

**********

4. Have the authors made all data underlying the findings in their manuscript fully available (please refer to the Data Availability Statement at the start of the manuscript PDF file)?

The PLOS Data policy

Reviewer #1: Yes

Reviewer #2: Yes

Reviewer #3: Yes

**********

5. Is the manuscript presented in an intelligible fashion and written in standard English?

Reviewer #1: Yes

Reviewer #2: Yes

Reviewer #3: Yes

**********

Reviewer #1: All corrections done.

Reviewer #2: (No Response)

Reviewer #3: Thank you for the revised version of the manuscript and for the detailed point-by-point response to the reviewers. In my view, the authors have adequately addressed the main methodological and reporting concerns raised in the previous rounds.

The manuscript now clearly defines the scope of the empirical evaluation. The authors state that the reported performance applies to the MIMIC-IV demo / EHRSQL 2024 setting, and they correctly leave the evaluation on the full MIMIC-IV dataset through BigQuery as future work. This distinction is important and is now sufficiently clear in the abstract, methods, discussion, and limitations.

The statistical reporting has also been improved. The authors provide Wilson 95% confidence intervals and use a paired McNemar exact test for the comparison between Claude Sonnet 4 and gpt-oss-20B on the same answerable questions. The interpretation is now appropriately cautious, as the manuscript reports no statistically detectable difference rather than claiming formal equivalence.

I also appreciate the clarification of the human evaluation protocol, including the use of a multidisciplinary panel, independent review by at least two evaluators, and consensus resolution. This improves the transparency of the scoring process. The unanswerable-question evaluation is now clearly presented as limited to gpt-oss-20B, and the authors no longer generalize this abstention result to Claude Sonnet 4.

The security wording has also been corrected. The manuscript now describes OAuth2/JWT, read-only SQL validation, output limits, rate limiting, and audit logging as design and implementation features, while explicitly stating that formal security testing remains future work. This is a more balanced and accurate presentation.

The data and code availability statement is acceptable. The authors specify the credentialed-access conditions for MIMIC-IV on PhysioNet and provide a public GitHub repository with the code, scripts, benchmark outputs, model responses, SQL queries, reference answers, and error-analysis materials.

Overall, I consider that the revised manuscript is technically sound, methodologically clearer, and appropriately limited in its conclusions. I do not have further substantive comments for the authors. I support publication.

**********

what does this mean?). If published, this will include your full peer review and any attached files.

**Do you want your identity to be public for this peer review?**  If you choose “no”, your identity will remain anonymous but your review may still be made public.

For information about this choice, including consent withdrawal, please see our Privacy Policy

Reviewer #1: **Yes:** Dr. Asim Mushtaq

Reviewer #2: No

Reviewer #3: **Yes:** Carlos Luis Parra-Calderón

**********
